# The Effect of the Traverse Feed Rate on the Microstructure and Mechanical Properties of Laser Deposited Fe_3_Al (Zr,B) Intermetallic Alloy

**DOI:** 10.3390/ma11050792

**Published:** 2018-05-14

**Authors:** Magdalena Łazińska, Tomasz Durejko, Tomasz Czujko, Zbigniew Bojar

**Affiliations:** Department of Advanced Materials and Technologies, Military University of Technology, Gen. Urbanowicza 2 Str., 00-908 Warsaw, Poland; magdalena.lazinska@wat.edu.pl (M.Ł.); tomasz.czujko@wat.edu.pl (T.C.); zbigniew.bojar@wat.edu.pl (Z.B.)

**Keywords:** laser deposition, iron aluminides, cooling rate, microstructure, precipitation

## Abstract

The results of the fabrication of components made with Fe-30%Al-0.35%Zr-0.1%B alloy powder using the Laser Engineered Net Shaping (LENS^TM^) system operated at different traverse feed rates are described in this paper. The temperature of the molten metal pool was recorded during this process. Depending on the assumed feed rate, the formation of Zr–based precipitates with various morphologies and distributions was observed in the structure of the investigated material. It was found that as the traverse speed increased, spheroidization, refinement, and a more homogeneous distribution of these precipitates occurred.

## 1. Introduction

The search for a new generation of materials that are resistant to high temperatures and aggressive environments is a driving force for extensive research in materials science and engineering conducted through the modification of both the chemical composition and structure of materials, as well as the use of new, advanced technologies. Nowadays, promising and innovative production methods for modern alloys include laser 3D metal printing techniques [1]. One such method is the rapid manufacturing of the final products (requiring only a finishing treatment), known as LENS^TM^ (Laser Engineered Net Shaping) [2]. This technology uses not only layer-by-layer reproduction of a CAD-designed product to precisely shape component geometries, but also advanced steering and controlling devices to obtain pre-assumed microstructural features, depending on the expected application of the material [3].

The LENS MR-7 model (laboratory systems provided by Optomec company, Albuquerque, NM, USA) device is equipped with a working table capable of moving in the XY plane and a laser beam head with a powder nozzle located above the table. Using four nozzles circumferentially spaced around the laser head, the powder is supplied to a focused laser beam, where it is melted and deposited on the substrate or a previous material layer. At the end of the deposition of each layer, the nozzle head is raised along the Z-axis with a value equal to the layer thickness. The process is carried out in a protective atmosphere where the level of oxygen and water vapor is less than 10 ppm [4]. Due to the precise control of the powder feed rate, laser power, travel speed and focusing position, the LENS technique provides a powerful tool for microstructure development of an as-built component. In the LENS system, a high-speed imaging technique (non-contact measurements) was used to study the deposition process and observe the focal molten zone [5,6]. These measurements (based on measuring the size of the molten pool) allow the determination of the temperature profile, temperature gradient and cooling rate in the vicinity of the molten pool. The cooling rate and solidification velocity at the solid–liquid interface of the molten pool during the deposition process influence the microstructure and mechanical properties of the final parts. It has been shown that a specified type of microstructural morphology (e.g., columnar structure, fine-grained or mixed) can be obtained in alloys shaped using the LENS technique through indirect control of the heat transfer rate by changing the feed rate of the working table (traverse speed) [5,7]. The LENS technique is currently applied for the manufacturing of components made from a wide range of materials, such as steel [8,9], titanium [10,11], ceramics [12,13], high-temperature materials [14], and high-entropy alloys [15,16]. Additionally, the LENS process allows deposition blends of elemental powders to create alloys and composites in situ [17,18,19,20]. Due to its specific characteristics, the laser-based LENS technique can also be considered an alternative method for producing and processing technologically difficult materials, such as Fe-Al intermetallic-based alloys [21]. These materials are competitive with conventional metallic materials at high temperatures and in highly corrosive environments [22]. Fe-Al intermetallics, including Fe_3_Al-based alloys, are an attractive alternative to the expensive high-temperature alloys that are currently applied. They are characterized by high resistance to oxidation, carburization and sulfidization at high temperatures, good strength, low density (~6 g/cm^3^), and low cost of raw material. Recently, interest in intermetallic-based protective coatings [23], foams [24,25] and gradient materials [26] has significantly increased. However, wide commercialization and implementation of this type of alloy in industrial practice is strongly limited, mainly by their low creep resistance above 600 °C [27]. Thus, current research on Fe_3_Al-based intermetallic alloys is focused primarily on increasing the working temperature range up to the 600 °C–900 °C range, i.e., above the stability of the D03 ordered cubic crystal structure. Based on the results presented in [28,29,30,31], improvements of the creep resistance of the analyzed alloys can be obtained by the following:

- solid solution strengthening through doping with a small amount of Cr, V, Mo or Ti,
- precipitation strengthening by alloying with Zr, Ta, or Nb or by increasing the content of C and B,
- coherent precipitation (A2 in B2 or A2 in B2 for Fe-Al-Ni-Cr alloys or B2 + L21 in Fe-Al-Ta alloys), or
- increasing the ordering (increased temperature stability, e.g., of the D03 phase in a Fe-Al-Ti alloy).

Additives such as Nb or Mo lead to improvements in the creep strength, but the greatest attention has been given to additions such as dispersed particles (e.g., borides and oxides), second-phase precipitates or intermetallic particles (e.g., Laves intermetallics) [32]. In particular, alloying additions, which can be retained in the solid solution in reasonable quantities at high temperatures after solidification, have fairly high partition coefficients between solid and liquid solubilities, and low solubility and diffusivity at low temperatures are sought. To improve the high-temperature properties of such materials, doped zirconium is often used [31]. The Zr addition also meets the specified selection criteria. The addition of a small amount of zirconium leads to the formation of the λ_1_-Laves phase Zr(Fe,Al)_2_ and/or τ_1_-phases Zr(Fe,Al)_12_ [33]. These Laves and τ_1_ phases increase the low creep resistance at high temperatures (and strength at high temperatures) of Fe_3_Al alloy. Kratochvíl et al. showed that the addition of Zr to Fe30Al leads to the formation of an Fe-Al (B2/D03) matrix and thin lamellae of the λ_1_-Laves phase Zr(Fe,Al)_2_, which increases the high temperature yield stress and strength [31]. Dobes et al. [34] showed that the creep resistance at 700 and 900 °C increases with the secondary phases λ_1_ and τ_1_. Nevertheless, the fine particles in alloys with low Zr content are able to restrict dislocation motion more effectively than coarse and distant particles in alloys with a high Zr content. However, the effect on creep resistance for an alloy with 5.2% Zr is comparable to that for an alloy containing 0.35% Zr and 0.1% B. Morris et al. observed that the Fe30Al0.35Zr0.1B alloy in the as-cast state during creep testing at 700 °C exhibited good creep resistance [30]. This good strength was associated with the precipitation of (Fe,Al)_x_(Zr,Al)_y_ particles, which were identified as the Fe_3_Zr or Fe_23_Zr_6_ phase. However, the results of a more detailed microstructural study have revealed that these particles are heterogeneously distributed, which has an adverse impact on the high temperature properties of these materials. The authors of some of these papers [31,34] suggest that a conventionally cast FeAl alloy with Laves phases has an inadequate distribution of the brittle intermetallic phase along the grain boundaries and interdendritic regions in the form of continuous films. They observed that the coarse and too-distant particles of λ and τ_1_ in Fe-Al-Zr alloy could not restrict dislocation motion. The search for an effective structural modification should therefore be conducted by optimizing the crystallization conditions, employing an additional heat treatment, or using another production method for this type of alloy. The alternative methods for producing Fe-Al alloys with controlled structure and morphology of the second phase are laser additive techniques. However, the investigations of iron aluminides obtained by additive manufacturing are rare, and most of the work concerns the binary Fe_3_Al phase [35,36,37,38,39,40,41,42]. Therefore, the authors of only a few papers [21,43,44,45,46,47] have addressed the subject of the complex compositions of Fe_3_Al-based alloys. Michalcová et al. [43] used selective laser melting (SLM) and laser metal deposition (LMD) technology to produce Fe30Al10Ti, Fe30Al5Ti0.7B and Fe22Al5Ti alloys, representing three different strategies for strengthening iron aluminides at high temperatures: increasing the ordering temperature D03↔B2, precipitation of borides, and generation of coherent A2 + L21 microstructures. Although the refinement of the microstructure (grain size of 5–10 µm) of these alloys was obtained, the ductility was not improved. As for application of LENS technology for manufacturing Fe-Al alloys, Durejko et al. [21] reported the successful fabrication of an Fe_3_Al (Fe30Al0.35Zr0.1B) alloy with low porosity and good mechanical properties (the strength at 650 °C matches that of the as-cast alloy) using the LENS system. Additionally, LENS technology has been employed to produce Fe28Al5Cr0.08Zr0.04B (at %) [44] and Fe28Al5Cr1Nb2B (at %) [45] alloys prepared from pre-alloyed gas-atomized powders. In the first case, the alloy was characterized by a very good oxidation resistance at high temperatures, which was comparable to or slightly better than that of the bulk materials produced using traditional technologies. FeAlCrNbB alloys were manufactured by varying the technological parameters [45]. The deposited material was characterized by a fine, equiaxed grain structure (grain size ~10 μm) over the entire volume of the sample and second-phase particles (NbB and NbB_2_) mainly arranged in the grain boundary areas. The authors showed that the boride particles contributed to a significant improvement in the compressive strength. In [46], Fe-Ti-Al alloy was fabricated from a mixture of gas-atomized Fe_3_Al and elemental Ti powders using LENS technology. The obtained single-phase (L21 Heusler phase), two-phase (L21 + C14 Laves phase) and three-phase (L21 + C14 + disordered A2 α-Fe,Al) alloys were characterized by a fine-grained microstructure (grain size in the range of 3–5 μm), but were inhomogeneous and exhibited macroscopic cracks and porosity. Recently, FeAl intermetallic-based alloys were manufactured by the LENS method using a mixture of FeAl alloy and nanometric Al_2_O_3_ powders [47]. The microstructural evaluation did not reveal any Al_2_O_3_ nanoparticles in the LENS fabricated alloy. However, it was found that the addition of 2 vol % n-Al_2_O_3_ significantly decreases the porosity (~1%) compared to the reference material (pure FeAl, ~5%), inhibits grain growth and improves oxidation resistance.

In the present work, the LENS technique was used to form microstructures (including the morphology of zirconium precipitates) of Fe_3_Al intermetallic alloy doped with boron and zirconium (Fe30Al0.35Zr0.1B) using various values of the technological parameters (different traverse feed rates).

## 2. Experimental Procedures

**Figure 1:**
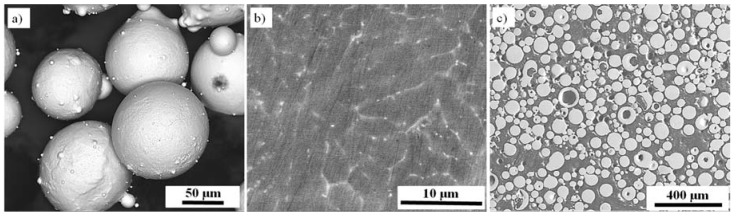
Initial Fe30Al0.35Zr0.1B powder (**a**) 3D view; (**b**) microstructure; and (**c**) metallographic cross section.

Spherical Fe30Al0.35Zr0.1B alloy powder (Figure 1a) (LERMPS, France) characterized by a normal distribution of particles with a diameter in the range of 44–150 μm was used as the input material for the manufacturing process described here. The microstructure of the input powder consisted of equiaxed grains of the Fe_3_Al intermetallic phase with an average diameter of 10 μm and continuous Zr-based precipitates located mainly along the grain boundaries (Figure 1b). The cross-section of the embedded powder particles (Figure 1c) demonstrates that the particles are porous with a pore volumetric fraction of approximately 3.6%.

The Fe_3_Al samples were prepared using a LENS MR-7 (Optomec, Albuquerque, NM, USA) laboratory system equipped with a fiber laser with a maximum power of 500 W. This device, through the use of numerical control and coupled interdependent movements of the working table in the XY working plane and a laser head along the Z-axis, gives the possibility of producing details using a powder mixture simultaneously supplied from a maximum of four powder feeders. A detailed description of the LENS system is provided in [21]. Additionally, the system is equipped with a thermal camera (ThermaVizTM) with a two-wavelength pyrometer (long band ranges from 850 nm to 950 nm, and short band ranges from 750 nm to 850 nm) and software designed for temperature monitoring during deposition of subsequent layers, as well as for plotting and analyzing the obtained temperature profiles. During sample fabrication, the temperature of the molten metal pool was recorded at a frequency of 100 Hz. The cooling rate during laser surface melting was estimated based on the following equation:

(1) $$\frac{dT}{dt}=\frac{VdT}{dx}$$

where *V* is the traverse speed [mm/s] and *dT*/*dx* is the temperature gradient.

Using the recorded temperature profiles, the cooling rate for each technological variant was determined. After loading the alloy powder feedstock, samples with dimensions of 3 mm × 6 mm × 30 mm were deposited on a previously blasted Armco iron substrate with a thickness of 11 mm. The samples were deposited layer by layer under the control of software that monitors the parameters, with a single deposited layer thickness of 0.15 mm. For each layer, a contour was first built along the perimeter of the sample and then the parallel hatches (using a zig-zag method) with 0.225 mm distance between them were deposited. The deposition lines for the next layer were deposited at an angle of 90 degrees to the previous ones and such process was repeated until a 3D sample was completed.

During preparation of the specimens with the geometry programmed via the CAD approach, the traverse feed rate and the powder feed rate were controlled with a constant 300 W laser power, and the temperature in the molten metal pool was recorded (Table 1).

During this process, the laser was focused on a metal substrate/the depositing layer of the parts, and the focal diameter was approximately 0.8 mm. The process was carried out in a controlled atmosphere of argon (the oxygen content was below 10 ppm). Additionally, argon was used as the carrier gas and as a shielding gas to protect the optical lens from powder intrusion and flares.

The obtained LENS samples were cut by electrodischarge machining perpendicular to the direction of layer deposition, then mechanically ground with progressively decreasing grit size SiC paper and polished with a 3 μm diamond suspension on a STRUERS PLANOPOL 3 machine (Struers Aps., Ballerup, Denmark). For metallographic observations of the microstructures using light-optical microscopy, the samples were etched with a mixture of 33%-CH_3_COOH, 33%-HNO_3_, 33%-H_2_O and 1%-HF. A quantitative analysis of the structural morphology obtained after different variants of the LENS process was carried out using a Nikon MA 2000 optical microscope equipped with an image analyzer and the NIS-Elements BR 3.2 software. An equivalent diameter (ECD) and a shape factor *α_k_* were expressed as follows:

(2) $$ECD=\sqrt{\frac{4\cdot P}{\pi }},$$

(3) $$\alpha_{k}=\sqrt{\frac{4\cdot \pi \cdot P}{\alpha^{2}}}$$

where *P* and *O* are the surface area and perimeter of the tested object, respectively. The obtained samples were also evaluated in terms of the heterogeneity of the distribution of Zr particles. According to the literature, it was assumed that a homogeneous structure is characterized by the variance (heterogeneity index) of the distance between adjacent objects. The heterogeneity index was calculated according to the equation:

(4) $$\xi =\left|\frac{\sigma_{j}^{2}-\sigma^{2}}{\sigma_{j}^{2}}\right|,$$

where *σ_j_*^2^ is the variation of the average distance between particles for a homogeneous microstructure (taken as 0.1) and *σ*^2^ is the variation of the average distance between particles for the real structure. The size of the Zr precipitates were determined based on binarized SEM images using an image analyzer and NIS-Elements BR 3.2 software (version 3.2, Nikon, Amsterdam, Netherlands).

An FEI Quanta 3D field emission gun (FEG) scanning electron microscope (SEM) with energy dispersive spectroscopy (EDS) was used to determine the chemical composition in micro-regions of the material, with a particular emphasis on the Zr-enriched particles. The chemical composition was measured using an EDS detector for three different areas, calculating the mean value and standard deviation. An electron backscatter diffraction (EBSD) system coupled with the FEI Quanta 3D field emission gun scanning electron microscope (FEG-SEM) was applied in order to analyze the complex microstructure. Before the examination, the samples were ground with 120–4000 grit SiC paper, polished with 3–0.25 μm diamond suspensions, and finally polished with 0.1–0.06 μm silica suspensions. For automatic crystal orientation mapping (the ACOM technique, also known as Orientation Imaging Microscopy, the TSL OIM Analysis 5.31 commercial software (EDAX, Mahwah, NJ, USA) was used.

The phase composition of the selected samples was determined by X-ray diffraction (XRD) analysis carried out on a Rigaku Ultima IV diffractometer, operated using a cobalt target, a 20°–140° angular range, a 0.02° step size and a 3 s dwell time.

The mechanical properties of the obtained samples were determined by Vickers microhardness testing and static compression tests at room temperature conducted on an Instron 8501 using a strain rate of 10^−4^ s^−1^. Vickers microhardness testing was performed on each sample using a SHIMADZU M machine (Kyoto, Japan) with a 100 g load and a dwell time of 5 s. For the compressive stress–strain tests, the cylindrical samples had a diameter of approximately 5 mm and a height of 7.5 mm and were fabricated by electro-discharge machining (EDM) (ZAPbp, Końskie-Kutno, Poland) parallel to the building direction. Surface layers were removed by grinding

## 3. Results and Discussion

**Figure 2:**
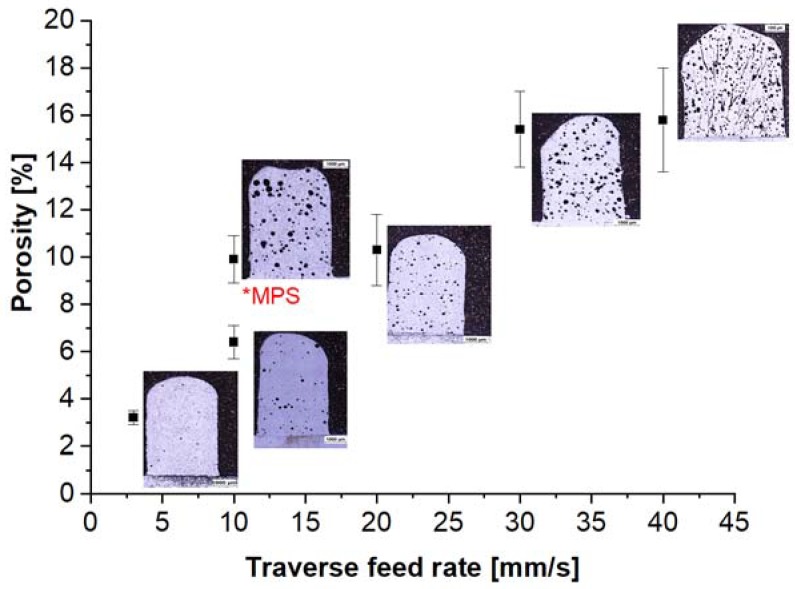
Influence of the traverse feed rate on the porosity of Fe_3_AlZrB alloys (*MPS-melt pool sensor—this is a control system monitoring the size of the liquid metal pool and providing feedback between the size of the pool and the power of the laser).

The microstructural observation showed that the obtained materials are characterized by the presence of spherical voids in all the variants. However, the degree of porosity depends on the working table feed rate (Figure 2). The porosity of samples increases with the deposition rate. For samples obtained at a deposition rate of 3 mm/s, the porosity is 3.2%, whereas for a sample obtained at a deposition rate of 30 mm/s, the porosity is as high as 15.8%. Due to the high deposition rate, the powder delivered into the laser focus spot is not fully molten (partially molten, which could initiate the formation of pores in the material), and the preset processing power is insufficient for the entire batch of powder to become molten in such a short period. Increasing the deposition rate causes shorter laser-particle interaction times. The short laser interaction time and low specific energy result in a high porosity level in the LENS fabricated sample. The smallest porosity was obtained for the longest laser interaction time of t_e_ = 0.27 s and the highest specific energy of E = 125 J/mm^2^. Additionally, for samples produced using the melt pool sensor software, an increase in porosity was observed compared to samples obtained using constant laser power settings.

Cracks were observed in the samples obtained at deposition rates of 30 and 40 mm/s. This was due to the high cooling rate and solidification cracking. In these variants, the time for laser-particle interactions was as short as 0.02–0.03 s at specific energies of 12.5 and 9.4 J/mm^2^ (Table 1). Every discontinuity, including instances of porosity, is an adverse factor, as it negatively affects the quality of materials. When the porosity is high, voids within the material volume may contribute to deterioration of the material resistance properties. Depending on their shape, the pores may lead to fracture by serving as a nucleation point. The authors of [21] suggest that the porosity of the LENS-fabricated Fe_3_Al components may be distinctly minimized by the proper selection of the process parameters: low powder flow rate and working table feed rate simultaneous with a high laser power. However, the complete elimination of porosity from the alloys is impossible due to porosity in the initial powder material. The porosity in the initial powder has a considerable impact on the metallurgical quality of the final sample [48]. The high porosity of the powder results in a high porosity in the deposit. Only a combination of parameters such as a low deposition rate and a high laser power can reduce the porosity in intermetallic alloys [21].

**Figure 3:**
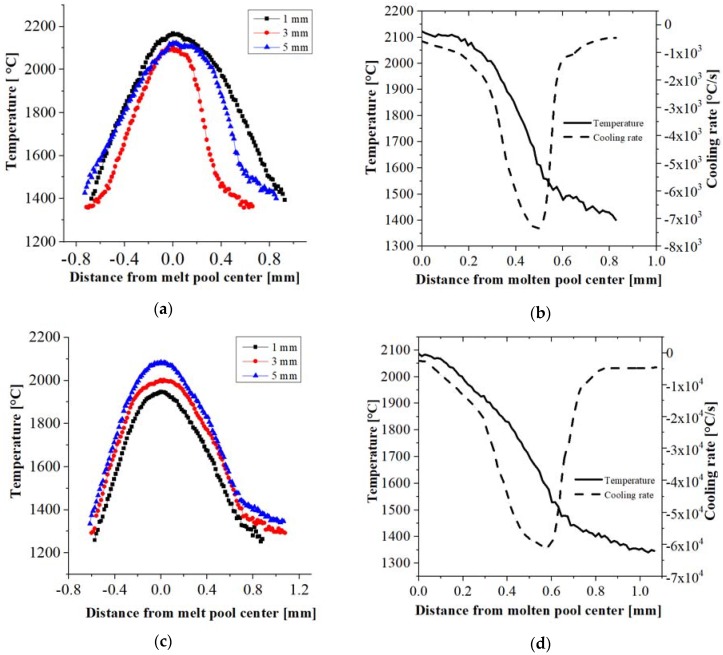
Profile temperatures and cooling rates for Fe_3_Al alloys manufactured with (**a**,**b**) 3 mm/s and (**c**,**d**) 30 mm/s traverse feed rates.

Based on the results of the temperature measurements of the molten metal pool for the various technological options, temperature profiles at distances of 1, 3 and 5 mm from the substrate were plotted. In each case, the liquid metal pool sizes are comparable (Figure 3a,c). It is also noted that the maximum temperature in the molten metal pool depends on the distance from the substrate. For samples produced with a 3 mm/s working table feed (Figure 3a), the highest temperature is recorded at a 1 mm distance from the substrate (2180 °C), whereas for the 30 mm/s feed, the maximum temperature (2100 °C) is observed at 5 mm (Figure 3c).

The resulting temperature profiles allow the determination of the cooling rate for each sample at a distance of 5 mm from the substrate (Figure 3b,d). It may be concluded that the cooling rate depends considerably on the rate of material deposition and, therefore, on the applied feed rate of the traverse. The use of a 3 mm/s feed value gives the smallest rate of heat transfer to the “cold” substrate among all the considered options, with its value at the crystallization front being ~7 × 10^3^ °C/s. A ten-fold increase in the deposition rate can increase the cooling rate by almost an order of magnitude to 6.1 × 10^4^ °C/s for a feed of 30 mm/s (Figure 3d). For intermediate feed values (10 mm/s and 20 mm/s), the heat dissipation rate reaches 1.6 × 10^4^ °C/s and 3.2 × 10^4^ °C/s, respectively (Table 2). The maximum considered feed (40 mm/s) results in an estimated heat transfer of 6.5 × 10^4^ °C/s.

**Figure 4:**
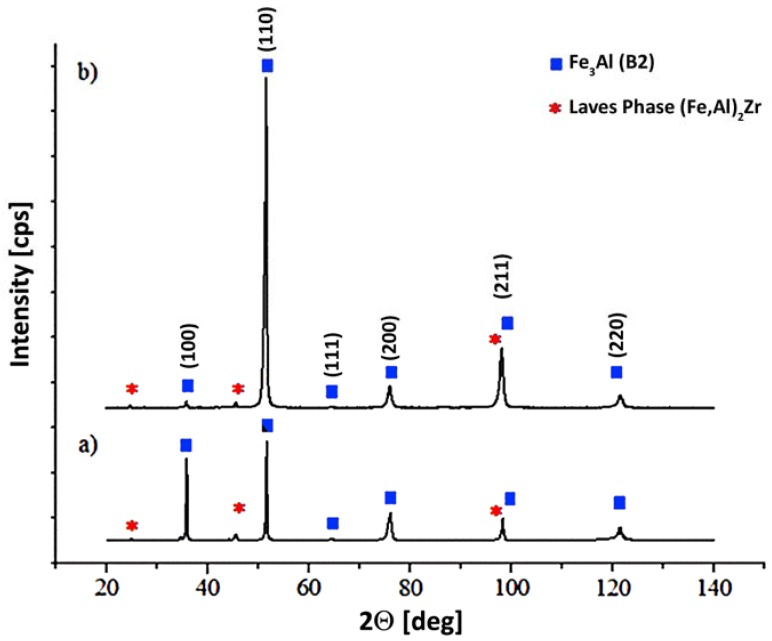
XRD analysis of the Fe_3_Al alloy fabricated at (**a**) 3 mm/s and (**b**) 20 mm/s traverse feed rates.

Due to the very small size of the precipitates, the estimation of the chemical/phase composition on the basis of the results obtained by the EDS method was difficult; therefore, XRD diffraction analysis was performed on the obtained samples. The analysis of the diffraction patterns reveals that the material (irrespective of the process variation and the working table feed) exhibits a long-range order that is specific to the B2-type superlattice structure (Figure 4), with no signs of the presence of D03. This phenomenon is typical for high cooling rates (10^4^ °C/s), such as that seen during LENS processing [21,45]. Simultaneously, reflections from the Laves phase are also observed and identified as an (Fe,Al)_2_Zr phase (Figure 4). A similar effect has been observed by Kratochvil [31] for the Fe30Al alloy with 0.4% Zr, where the particles identified as (Fe,Al)_2_Zr formed a lamellar eutectic with the Fe-Al (D03/B2) matrix.

**Figure 5:**
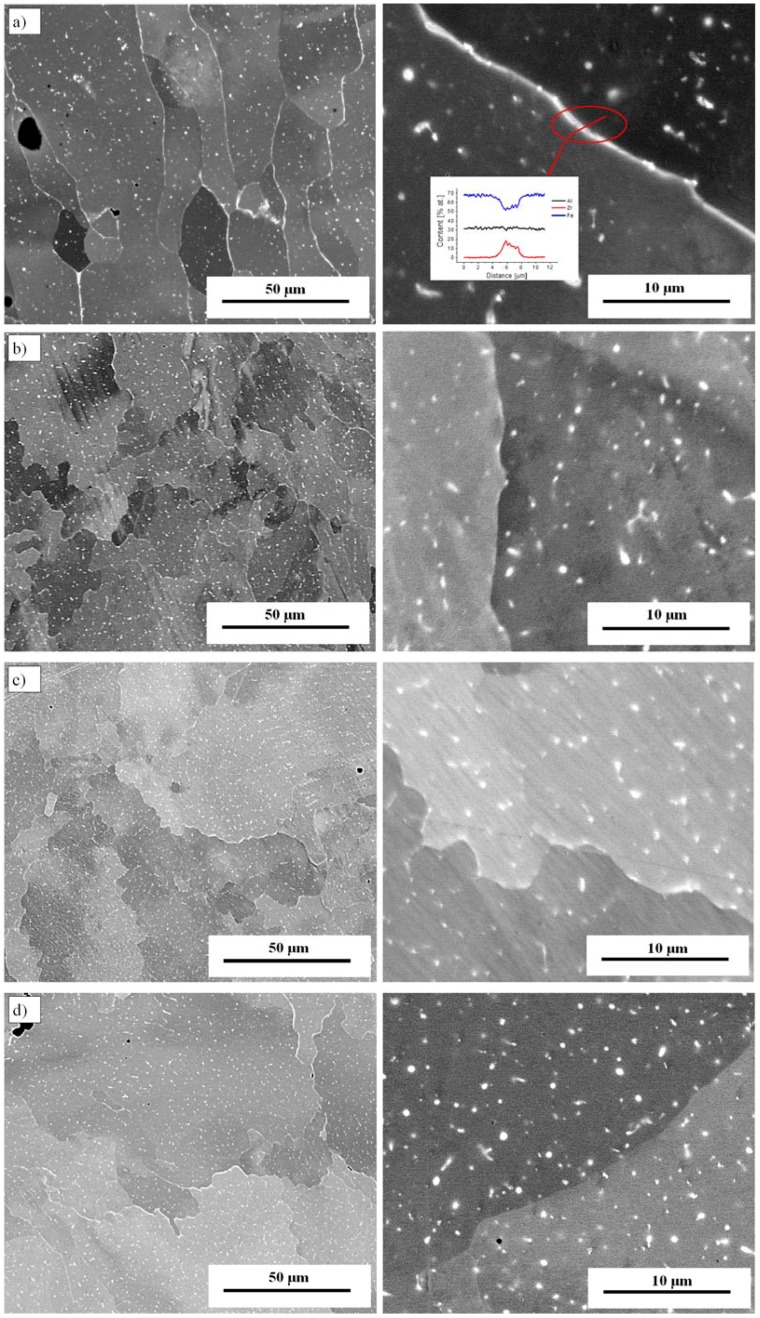
Microstructure of the Fe_3_Al alloy with Zr addition—traverse feed rates: (**a**) 3 mm/s; (**b**) 10 mm/s; (**c**) 10 mm/s with MPS; and (**d**) 20 mm/s.

Results from the metallographic analysis performed for all the considered technological variants indicate that the traverse feed rate has a significant impact on the morphology of both the Fe_3_Al grains and the zirconium-based precipitates. High zirconium content areas, regardless of the scanning speed, are located both along the grain boundaries and in the volume of the Fe_3_Al grains (Figure 5). The EDS analysis gives average values of 30.32 ± 0.5 at % Al, 0.39 ± 0.03 at % Zr and 69.29 ± 0.3 at % Fe in the alloy matrix. The values are similar to those of the initial powder (Fe30Al0.35Zr0.1B at %). An increase in the zirconium content is observed (Figure 5a).

For the first variant (with a 7 × 10^3^ °C/s cooling rate), the Zr-based precipitates are found mainly in a continuous form along the boundaries of the Fe_3_Al grains (Figure 5a). Changing the parameters of the manufacturing process enables the efficient refinement of zirconium precipitates, particularly those located at boundary areas.

For an average feed (3 mm/s), the zirconium precipitates form continuous, compact structures in the grain boundaries of the Fe_3_Al grains (Figure 5a). With an increase in the feed value, these microstructural features undergo intense fragmentation and refinement (Figure 5b).

The microstructure of the obtained material was subjected to detailed quantitative studies. Fe_3_Al grains and zirconium precipitates were analyzed in terms of their size and shape using measurements of such parameters as the equivalent diameter and the shape aspect ratio. On the basis of the conducted structural analysis (Table 2), it can be concluded that there is a strong relationship between the applied feed of the working table and the obtained size of the Fe_3_Al grains. For all the analyzed cases, the aspect ratio deviates significantly from unity. This is evidence of the presence of a non-equiaxed grain structure, which, in particular for the 6th variant (Table 2), exhibits a highly elongated shape associated with the direction of heat dissipation. Moreover, for this case, the largest grain equivalent diameter (~90 μm), with high standard deviation, was also reported. The highest refinement and structural homogeneity were observed for the 5th variant, which was obtained using automatic laser power control.

From the point of view of the planned high-temperature applications of the investigated alloy, precipitates or particles that strengthen the structure and increase the creep resistance play an important role. The proper selection of the manufacturing parameters, especially the working table feed, hints at the possibility to precisely control the morphology and uniformity of the distribution of zirconium particles/precipitates in an Fe_3_Al intermetallic alloy. The results of the stereological analysis of the strengthening phase morphology show that the desired results are obtained only for those variants where the feed is above 10 mm/s (Table 2).

In these cases, the zirconium-based particle/precipitate size is approximately 300 nm, with different aspect ratios for each individual case. Precipitates/particles with shapes most similar to spherical particles are obtained for the first and second variants. However, the average diameter of these particles is relatively large (Table 2). The resulting structure was also evaluated in terms of the heterogeneity of strengthening particle distribution. According to the literature, it is assumed that a homogeneous structure is characterized by the variance (heterogeneity index) of the distance between adjacent objects at a level below 0.1. The highest structural homogeneity, in terms of the distribution of the strengthening phase, is obtained for variants with a deposition rate greater than 10 mm/s. The value adopted for the evaluation of the homogeneity factor is close to 0.1 for these cases. Based on the obtained results, it may be stated that the LENS technique allows for the precise shaping of the structure, and the morphology of the particles/precipitates in particular, which, according to the literature data can have an impact on the high-temperature behavior of Fe_3_Al-based intermetallic alloys [34].

**Figure 6:**
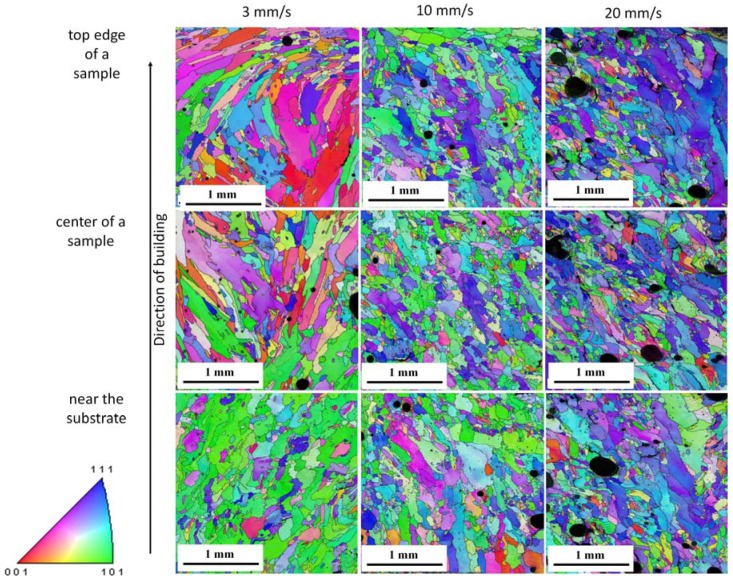
SEM EBSD orientation mapping of Fe30AlZrB obtained from different areas (near the top edge of a sample, in the middle part (center) of a sample, near the substrate (bottom)).

The EBSD analysis (Figure 6) shows that for all the samples, the grain size is not uniform. Both columnar and fine grains are observed. The grains show no preferred orientation (for the entire volume of the sample) and no crystallographic misorientations within the individual grains. The preferred orientation grain growth in the direction of (101) was observed only for the area near the substrate for the sample fabricated at 3 mm/s.

However, the traverse feed rate influences the grain size. For a sample obtained at a feed rate of 3 mm/s, the formation of finer grains in the first layers (near the substrate) is observed. From there, grains elongated to several micrometers in length (opposite to the cooling direction) are observed in the center of the sample and at the edge of the sample. A similar effect was observed for an Fe28Al alloy [38]. In the LMD and SLM samples, the few first layers were finer grained (lower region), and then, during the further build-up of the sample, elongated grains (approximately 900 µm) were observed. As the feed rate increases, the zone near the substrate tends to exhibit the growth of columnar grains (Figure 6). In contrast to the samples obtained at a feed rate of 3 mm/s, in the center and upper regions, a fine-grained structure exists. This is consistent with previous work [21,47] and is characteristic of the LENS fabrication process. The high temperature gradient and high cooling rate cause rapid heat dissipation and lead to rapid cooling via the substrate, which consequently promotes directional grain growth in this region of the sample.

**Figure 7:**
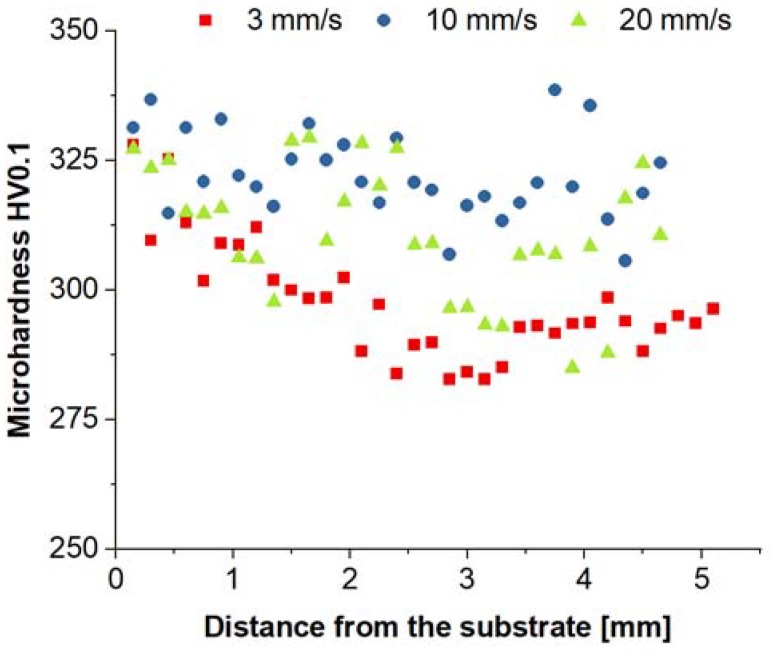
The microhardness distribution in the as-fabricated samples.

Due to the high porosity and cracks in the samples obtained at flow rates of 30 mm/s and 40 mm/s, mechanical properties were examined only for samples obtained at 3, 10, and 20 mm/s rates. Microhardness measurements were performed to compare the structural homogeneity of the samples (Figure 7). For samples obtained at the flow rate of 3 mm/s, the mean microhardness value is 297 ± 10 HV0.1. Slightly higher values of 322 ± 8 HV0.1 and 311 ± 12 HV0.1 are recorded for flow rates of 10 mm/s and 20 mm/s, respectively. The observed microhardnesses of the LENS samples are very similar to those for the LMD-processed Fe28Al (at %) samples (313 ± 13 HV0.1) with an average grain size of 300 μm [38]. An increase in the hardness is observed near the base of the samples. Upon manufacturing of elements using the LENS technique, the highest strains are generated near the substrate and gradually decrease toward the top layer [5]. The highest heterogeneity of the hardness values is obtained for the sample obtained at a flow rate of 20 mm/s due to the high porosity of the sample.

**Figure 8:**
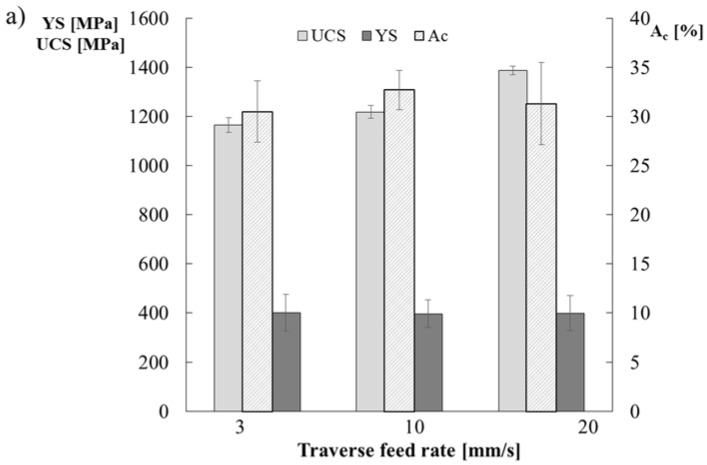

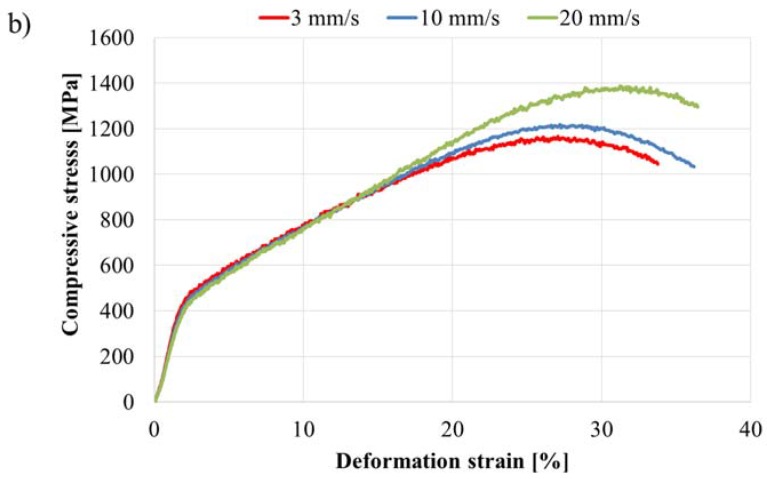
Mechanical properties (**a**) YS, UTS; Ac and (**b**) stress-strain curves of the Fe_3_AlZrB-based alloys fabricated with traverse feed rates of 3 mm/s, 10 mm/s and 20 mm/s.

The yield strength YS, the ultimate compressive strength UCS and the deformation strain A_c_ were determined from compression tests (Figure 8).

Based on these results, the traverse feed rate has no effect on the yield strength. For all the analyzed samples, the yield strength is estimated at YS = 400 MPa upon a deformation strain A_c_ of approximately 30%. Similar values are obtained for the cast alloy Fe30Al5Ti0.7B (460 MPa), as well as for the laser melt-deposited alloy Fe28Al (at %) being compressed in the direction perpendicular to deposition (400 MPa). A higher yield strength of 540 MPa is observed for the parallel direction and 490 MPa for the perpendicular direction for Fe30Al10Ti alloys obtained using the LMD method. The highest compression strength of 1387 MPa is obtained for a sample obtained at a flow rate of 20 mm/s, whereas the lowest compression strength of 1165 MPa is obtained for a sample obtained at a flow rate of 3 mm/s. All the samples were destroyed when the maximum compression strength was reached, with the cracks angled ca. 45° to the axis and appearing at the lateral surface of the samples in a manner characteristic for brittle materials.

## 4. Conclusions

In this work, the effect of the working table feed on the microstructure and properties of Fe30AlZrB alloy was investigated in detail. Based on the obtained results, it is clear that the LENS technique allows for precise shaping of the material microstructure through the proper selection of process parameters, which significantly affect the heat transfer rate. The temperature was recorded in the molten metal pool using a ThermaViz system, which allows indirect determination of the cooling rate and correlation with the obtained microstructure. The results indicate that the LENS method is particularly useful for non-conventional alloys based on intermetallic phases of the Fe-Al system, which is designed for operation at elevated temperatures. It not only allows fast development of simple or complex geometries, but also provides the ability to precisely control the morphology of structural components. Due to local, precise control of the crystallization process, the morphology of the second-phase precipitates can be shaped, which, in the case of Fe_3_Al alloy, is extremely important due to the poor creep resistance at elevated temperatures. The presence of zirconium precipitates uniformly distributed in the volume with simultaneous control of the morphology of the matrix grains creates new opportunities for the development and commercialization of bulk, hard Fe_3_Al alloys with particular attention paid to their working conditions. The main conclusions are formulated as follows:

- The working table feed significantly affects the porosity of the samples. When the speed increases, the degree of porosity increases. Furthermore, cracks are observed in the samples for a feed rate greater than 20 mm/s.
- Irrespective of the process variation, the analysis of diffraction patterns revealed that the material exhibits a long-range order specific for a B2-type superlattice structure. Reflections from the Laves phase were also observed and were identified as an (Fe,Al)_2_Zr phase.
- As the working table feed increased from 3 to 20 mm/s, zirconia fragmentation was observed with a slight change in grain size. The grains have a similar orientation in each of the samples.
- The working table feed does not affect the microhardness and yield strength of the Fe_3_Al samples.

## Figures and Tables

**Table 1:** Technological parameters associated with the Fe_3_Al sample manufactured using the LENS method.

Number of Samples	Traverse Feed Rate [mm/s]	Powder Feed Rate [g/min]	Specific Energy ** [J/mm^2^]	Laser/Particle Interaction Time *** [s]
1	10	3.4	37.5	0.08
2	20	8.5	18.8	0.04
3	30	12.5	12.5	0.03
4	40	16.1	9.4	0.02
5 (with MPS *)	10	3.4	37.5	0.08
6	3	0.8	125.0	0.27

* The sample was created using the MPS (melt pool sensor—this is a control system monitoring the size of the liquid metal pool and providing feedback between the size of the pool and the power of the laser); ** energy delivery per unit area of material, E = P/(2r_b_V_beam_), P—laser power, V_beam_—scan speed, r_b_—beam radius; *** exposure time, t_e_ = 2r_b_/V_beam_.

**Table 2:** Influence of the technological parameters on the Fe_3_Al grain morphology and Zr particle/precipitate morphology.

Sample Number	Cooling Rate (°C/s)	Average Equivalent Diameter of Grain Size (µm)	Shape Factor of Grain	Inhomogeneity of Zr Precipitate Distribution	Average Equivalent Diameter of Zr Precipitates (nm)	Shape Factor of Zr Precipitates
1	1.6 × 10^4^	78.51 ± 27.78	0.50 ± 0.16	0.28	432 ± 198	0.89 ± 0.12
2	3.2 × 10^4^	71.13 ± 21.33	0.58 ± 0.16	0.13	307 ± 185	0.67 ± 0.19
3	6.1 × 10^4^	70.52 ± 23.28	0.61 ± 0.15	0.12	306 ± 158	0.65 ± 0.17
4	6.5 × 10^4^	63.90 ± 21.94	0.63 ± 0.13	0.15	325 ± 153	0.71 ± 0.19
5	1.3 × 10^4^	59.71 ± 19.62	0.66 ± 0.15	0.27	293 ± 141	0.88 ± 0.16
6	7.2 × 10^3^	90.21 ± 31.12	0.40 ± 0.17	0.23	408 ± 187	0.93 ± 0.11
